# Swept-source OCT reduces the risk of axial length measurement errors in eyes with cataract and epiretinal membranes

**DOI:** 10.1371/journal.pone.0257654

**Published:** 2021-09-22

**Authors:** Francesco Faraldi, Carlo Alessandro Lavia, Marco Nassisi, Raphael A. Kilian, Daniela Bacherini, Stanislao Rizzo

**Affiliations:** 1 Surgical Department, Ophthalmology Service, Azienda Sanitaria Locale TO5, Chieri, Italy; 2 Ophthalmological Unit, Fondazione IRCCS Ca’ Granda, Ospedale Maggiore Policlinico, Milan, Italy; 3 Department of Clinical Sciences and Community Health, University of Milan, Milan, Italy; 4 Ophthalmic Unit, Department of Neurosciences, Biomedicine and Movement Sciences, University of Verona, Verona, Italy; 5 Department of Neurosciences, Psychology, Drug Research, and Child Health, Eye Clinic, University of Florence, AOU Careggi, Florence, Italy; 6 Ophthalmology Unit, Università Cattolica del Sacro Cuore, Rome, Italy; 7 Fondazione Policlinico Universitario A. Gemelli, IRCCS, Rome, Italy; 8 Consiglio Nazionale della Ricerca (CNR), Pisa, Italy; Nicolaus Copernicus University, POLAND

## Abstract

**Aims:**

To compare the biometric data from partial coherence interferometry (PCI) and swept-source OCT (SS-OCT) in patients with age-related cataract and epiretinal membrane (ERM): ERM, ERM with foveoschisis and macular pseudohole.

**Methods:**

49 eyes of 49 subjects including 36 ERM, 9 ERM foveoschisis and 4 macular pseudohole were analysed to evaluate the axial length (AL) measurements and the presence of AL measurement errors, defined basing on the shape of the biometric output graphs and on the concordance of AL values between instruments. Eyes with ERM were divided in four stages according to OCT features (i.e. presence/absence of the foveal pit, presence of ectopic inner foveal layers, disrupted retinal layers).

**Results:**

The devices provided similar mean AL measurements in all subgroups, with differences <0.1 mm in 41/49 cases (83.6%). AL measurement errors were observed in ERM stages 3 and 4, characterized by ectopic inner foveal layers, and were significantly more frequent with the PCI (8/17, 47%) as compared with the SS-OCT device (2/17, 12%), p = 0.02. The refractive prediction error in cases with AL measurement errors was significantly greater using the PCI compared to the SS-OCT device (p<0.05).

**Conclusion:**

Both devices provide reliable biometric data in the majority of patients and can be used in the preoperative assessment of patients with age-related cataract and ERM. In eyes with ectopic inner foveal layers, attention should be paid as AL measurement and refractive prediction errors may occur, more frequently with the PCI device.

## Introduction

Surgical procedures for vitreoretinal interface (VRI) disorders are nowadays more frequently performed on patients presenting with a relatively good visual acuity (i.e. 20/32), with a major need for optimal refractive and visual acuity outcomes [1, 2].

When a combined procedure (i.e. cataract and vitreoretinal surgery) is chosen, a correct intraocular lens (IOL) power calculation is among the main determinants of a satisfactory post-operative refraction [3, 4]. Moreover, with the increased use of premium IOLs (e.g. toric IOLs) even in patients with VRI disorders, the post-operative refraction has become crucial [5, 6].

In patients with VRI disorders, incorrect IOL power calculations are more commonly driven by errors in axial length (AL) measurement, as previously reported [7]. It is the presence of any epiretinal material (e.g. epiretinal membrane, thick posterior hyaloid), whose reflectivity may reduce or imitate that of the retinal pigment epithelium (RPE), that may prevent a correct AL estimate. Both ultrasound and optical biometers have been previously evaluated in patients with VRI disorders, with documented underestimations in AL measurements in those cases presenting epiretinal material [8].

New swept-source devices have been developed to analyse the anterior segment and to provide corneal, anterior chamber and AL measurements, with the potential advantage of a higher tissue penetration, faster acquisition, greater depth of field and image quality.

The Anterion swept-source optical coherence tomography (SS-OCT; Heidelberg Engineering, Germany) has been recently introduced and the accuracy and repeatability of its results have been tested with promising results [9, 10].

Aim of the present study is to compare the biometric data in patients with age-related cataract and VRI disorders using a partial coherence interferometer (PCI) and a SS-OCT.

## Materials and methods

This study, conducted in the Ophthalmology unit of Santa Croce Hospital, Moncalieri, Italy, was approved by the Ethics Committee (protocol number 155/2020, general registry number n°11486 4th September 2020, Inter-Hospital Ethics Committee, San Luigi Gonzaga Hospital, Orbassano, Italy) and adhered to the tenets of the Declaration of Helsinki. Informed consent was obtained from all subjects.

Subjects’ inclusion criteria were the presence of age-related cataract and VRI disorders including epiretinal membrane (ERM), ERM with foveoschisis and macular pseudohole presenting an indication for combined cataract and vitreoretinal surgery.

**Fig 1:**
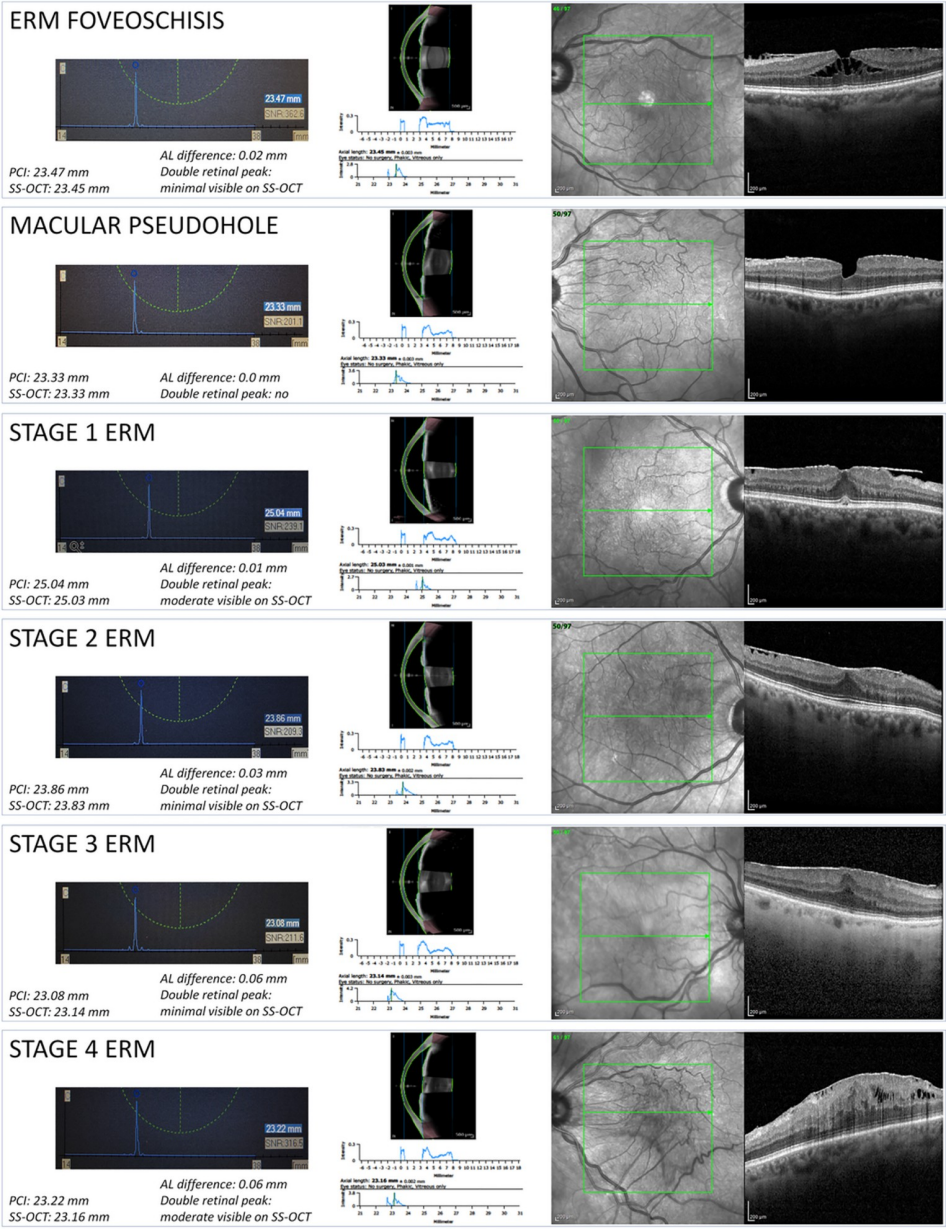
Subgroup biometric outputs and OCT scans. The six rectangular boxes represent the six subgroups analysed in the study, as reported in the captions (top left). Left part: in the light blue rectangles are reported the biometric outputs (x-y graphs) from the PCI device. The axial length (AL) in mm is reported on the x-axis, the signal amplitude is reported on the y-axis. The peak of the blue line is normally placed at the retinal pigment epithelium (RPE) level. The small blue rectangle on the right reports the mean AL. The small white rectangle on the right reports the signal-to-noise ratio (SNR). Middle part: anterior segment SS-OCT scan (top), graph reporting the signal intensity throughout the anterior segment (middle) and the biometric outputs (x-y graphs) from the SS-OCT device (bottom). In the biometric output the AL in mm is reported on the x-axis, the signal amplitude is reported on the y-axis. The blue line may present a single or double retinal peak. When present, the first retinal peak is located at the vitreoretinal interface, while the second peak is located at the RPE level. Right part: infrared image of the posterior pole (left) and horizontal OCT B scan (right) passing through the fovea. Left part, bottom: PCI and SS-OCT mean AL measurements. Left part, middle: AL differences between the devices and shape of the biometric outputs; when present, a double peak was described as minimal (intensity below < 50% of the second peak), moderate (between 50% and 100%), significant (> 100%).

Epiretinal membranes, ERM with foveoschisis and macular pseudohole were classified according to the International Vitreomacular Traction Study (IVTS) group and to a recent consensus [11, 12]. ERM were furthermore classified according to OCT appearance as proposed by Govetto et al [13] (Fig 1). In brief, the authors classified the ERM considering the following OCT features: stage 1 was characterized by the presence of the foveal pit, stage 2 by its absence while stages 3 and 4 by the presence of ectopic inner foveal layers with well-defined (stage 3) or disrupted (stage 4) retinal layers.

The indication for surgery was given by the surgeon (FF) basing on symptoms (metamorphopsia), visual acuity and clinical findings (degree of cataract, age, OCT features).

Patients who underwent previous ocular surgery or suffered from other retinal diseases (e.g. age-related maculopathy, pathologic myopia, glaucoma, uveitis, retinal vascular diseases) were excluded. Patients presenting opaque media (e.g. corneal leucoma, mature or subcapsular cataract) or poor fixation stability (e.g. nystagmus) reducing the reliability of acquired data were also excluded.

### Axial length calculation and analysis

IOL Master 500 (Zeiss, Germany) and Anterion SS-OCT examinations were performed before pupil dilatation. Axial length measurements were performed in both eyes using the IOL master 500 and Anterion SS-OCT devices.

IOL Master examinations with a sound-to-noise (SNR) value < 100 and Anterion SS-OCT examinations with a “fail” report were recorded but excluded from the analysis.

The IOL master 500 provides data on white-to-white distance, corneal curvature, anterior chamber depth and AL, as previously described [14]. In the current study, the AL was the outcome of interest that is, in brief, obtained by partial coherence interferometry, based on the Michelson interferometer using a laser diode that generates infrared light (λ = 780 μm) of short coherence length (CL = 160 μm). The system allows precise measurements of distances between the corneal and retinal interfaces, independent of longitudinal eye movements.

Anterion SS-OCT uses a 1300 nm light source, providing an axial resolution lower than 10 μm, a lateral scan angle up to 16.5 mm wide and a scan depth range of 14±0.5 mm. The wavelength of the Anterion SS-OCT allows a complete, all-in-one evaluation of the anterior segment. The device contains 2 imaging modalities, a lateral scanning SS-OCT and an infrared camera. The first one is used for cross-sectional imaging providing data on corneal thickness and curvature, corneal aberrations, aqueous depth, lens thickness, anterior chamber volume and AL; the second one allows an en-face imaging of a subject’s eye showing pupil characteristics [15].

Both instruments provide an AL measurement from the corneal surface to the RPE. The mean AL measurement values, as reported by each device as the resultant of several consecutive measurements, were recorded for each patient.

Both devices provide an x-y graph showing the intensity peaks (placed on the RPE) used to calculate the axial length.

The RPE peaks could be influenced in shape and width by several factors like opacities (e.g. corneal, lenticular, vitreous) and hyper-reflective material (e.g. epiretinal material, vitreomacular tractions). In case of epiretinal material, a double peak could be observed, with the first at the ERM level and the second at the RPE level.

In this study, to evaluate the incidence of AL measurement errors, we performed a case-by-case analysis considering the reported AL obtained with each device, resulting in different scenarios:

- Equal / similar (i.e. within 0.1 mm) AL obtained with the two devices;
- Different AL obtained with the two devices

**Fig 2:**
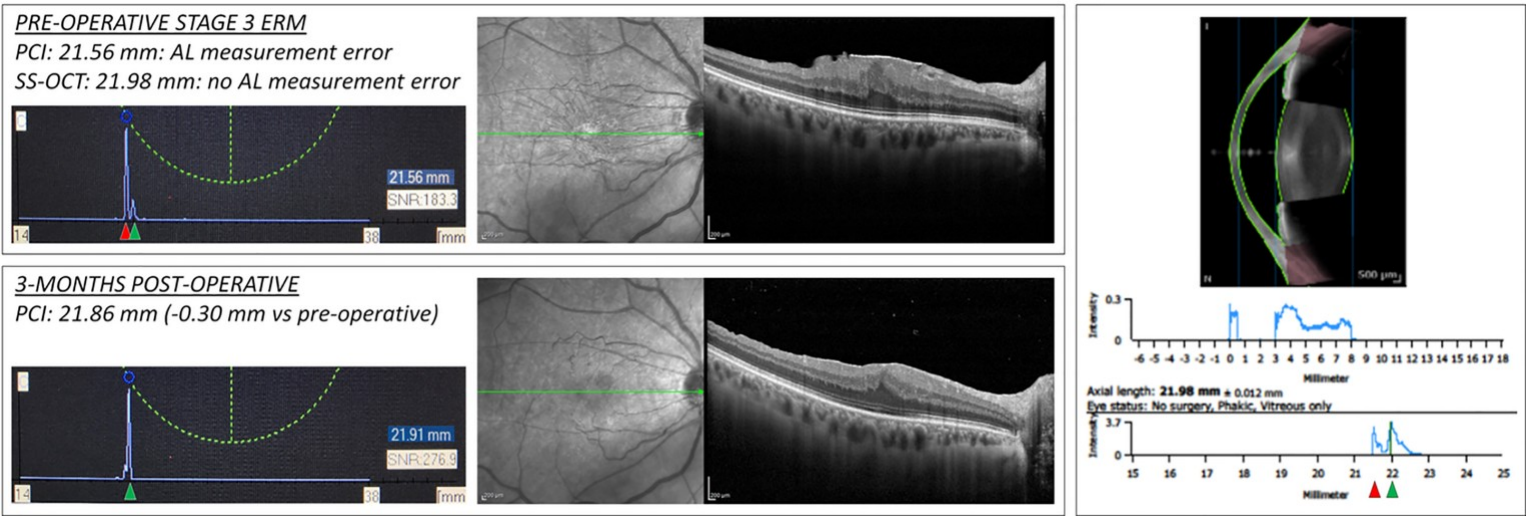
AL measurement error of the PCI device in a case of ERM stage 3. Left part, upper box: PCI biometric output in a case of ERM stage 3, as shown in the OCT B-scan on the right. The measured mean AL is 21.56 mm, as reported in the caption and in the blue rectangle. Left part, lower box: PCI biometric output of the same eye 3 months after combined cataract and vitreoretinal surgery with ERM removal. The measured mean AL is 21.91 mm. Right part: anterior segment SS-OCT device output. The measured mean AL is 21.98 mm. The pre-operative AL measurement obtained with the PCI was considered erroneous. In the pre-operative PCI biometric output, a double peak is present (first: red arrowhead; second: green arrowhead). After surgery, a single peak is present (green arrowhead), likely corresponding to the second smaller peak visible in the pre-operative output. The first peak in the pre-operative biometric output likely corresponds to the hyper-reflective vitreoretinal interface and the second to the RPE. In the SS-OCT biometric output, a moderate double peak is visible, with the first likely representing the vitreoretinal interface (red arrowhead) and the second the RPE (green arrowhead).

**Fig 3:**
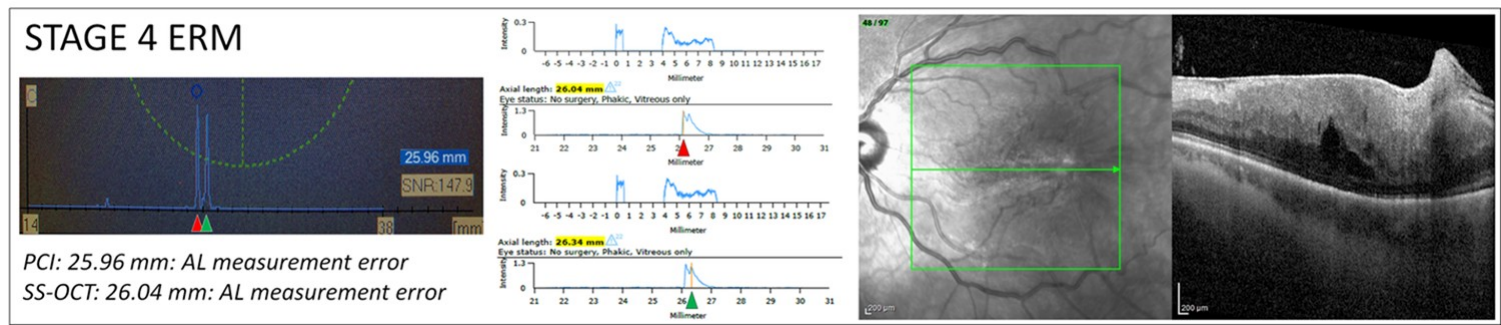
AL measurement error of the PCI and SS-OCT devices in a case of ERM stage 4. Left part: PCI biometric output in a case of ERM stage 4, as shown in the OCT B-scan on the right. The measured mean AL is 25.96 mm. Middle part: SS-OCT biometric output of the same eye with two different measurements. Top: the mean AL, measured by moving the cursor on the first peak, is 26.04 (highlighted in yellow); bottom: the mean AL, measured by moving the cursor on the second peak, is 26.34 (highlighted in yellow). The first peak presents greater signal intensity compared to the second. AL measurements from both devices were considered erroneous. In the PCI biometric output, a double peak is present with two distinct peaks presenting similar signal intensity. In the SS-OCT biometric output a significant double peak is visible. The first peaks (red arrowheads) likely correspond to the vitreoretinal interface. The second peaks (green arrowheads) may correspond to the RPE or outer retinal structures.

Then, considering the AL data, we performed an analysis of the x-y graphs, resulting in different scenarios:

- Single peak with equal / similar AL obtained with the two devices: AL measurement error unlikely (in case, regarding both devices; example in Fig 1)
- Single (or double) peak with different AL obtained with the two devices: AL measurement error possible (in case, regarding one device; example in Fig 2)
- Double peak with equal / similar AL obtained with the two devices: AL measurement error possible (in case, regarding both devices; example in Fig 3)

In presence of a double peak, an AL measurement error occurs when the first peak (at the ERM) is higher than the second (at the RPE), resulting in an underestimation of the AL.

The Alcon single-piece IOL AcrySof SN60WF (Alcon Laboratories, Inc.; A-constant 118.7 for optical SRK-T) was chosen for implantation at the end of cataract surgery. The Hoffer Q, SRK-T and Holladay I formulas for IOL power calculations were chosen according to AL and manufacturers recommendations. For each case, the same formula was used with both devices.

### OCT analysis

Spectral-Domain OCT (SD-OCT) examinations were performed with dilated pupils. In all subjects, a horizontal raster acquisition of ≈ 20x20° centred on the fovea, composed by 97 parallel B-scans was acquired. Adjunctive scans were performed in areas of interest when deemed necessary.

OCT features were analysed by two expert examiners (FF and CL) and used for group and subgroup classification.

### Statistical analysis

Continuous variables were checked to meet the normality conditions using the Shapiro–Wilk test. A parametric t-test or a Wilcoxon test was used when deemed necessary to compare the variables between groups. Statistical analysis was performed using IBM SPSS Statistics (SPSS Statistics, version 19.0, Chicago, IL, USA). Binary variables were arranged in cross-correlation tables and studied using the chi-squared test.

Results are presented as the mean ± standard deviation (SD) or as the median with range for continuous variables, and as proportions (%) for categorical variables. P-values < 0.05 were considered statistically significant.

## Results

Fifty-five consecutive patients were enrolled in the study. Six eyes were excluded from the study due to a SNR < 100 with the PCI (four cases), “fail report” with the SS-OCT (one case) or both of them (one case).

Forty-nine eyes from 49 patients (mean ± SD age 72.6 ± 5.7 years, range 62–89 years) were finally included in the study.

Epiretinal membrane were observed in thirty-six patients, ERM with foveoschisis in nine patients and macular pseudohole in four patients.

Overall, the mean ± SD AL was 23.73 ± 0.98 mm (range 21.56–26.44 mm) using the PCI device and 23.75 ± 0.97 mm (range 21.98–26.45 mm) using the SS-OCT. There was no significant difference in AL measurements between the two devices (p = 0.89).

Overall, the devices reported a difference in AL measurements < 0.1 mm in 41/49 cases (83.6%).

The analysis of the AL measurement outputs revealed eight cases of AL measurement errors using the PCI device and two cases using the SS-OCT device. In all cases a thick posterior hyaloid or an epiretinal membrane were erroneously considered as the RPE, resulting in an underestimation of the AL measurement (Figs 1–3).

Subgroup analysis showed no significant AL measurement differences between the devices in the ERM with foveoschisis and macular pseudohole groups (Fig 2). The analysis of the AL measurement outputs from both devices revealed no AL measurement errors, with the retinal peaks correctly placed at the RPE level.

Subgroup analysis showed no significant AL measurement differences between the devices in the ERM group. The analysis of the AL measurement outputs revealed eight AL measurement errors with the PCI device and two with the SS-OCT device.

According to the classification from Govetto et al, the ERM group was furtherly divided in 4 groups, with ten patients with ERM stage 1, nine with stage 2, nine with stage 3 and eight in stage 4 [13].

Subgroup analysis of the ERM groups showed no significant AL measurement differences between the devices in each group. However, the analysis of the AL measurement outputs revealed a significantly higher number of AL measurement errors with the PCI device as compared with the SS-OCT device in eyes with ERM stage 3 (three versus one error with the PCI and SS-OCT device, respectively) and stage 4 (five versus one error with the PCI and SS-OCT device, respectively), p = 0.02. No AL measurement errors were observed in eyes with ERM stage 1 and 2.

### Patients with AL measurement errors

The eight patients in groups 3 and 4 presenting AL measurement errors underwent uneventful combined cataract surgery and vitrectomy; the IOL power was chosen on the basis of the measurement obtained 0with the SS-OCT device, after checking for the correct positioning of the RPE peak (as described in Figs 2 and 3). Three months after surgery, the mean uncorrected visual acuity was 0.25 ± 0.09 LogMAR, with an absolute mean postoperative manifest refraction spherical equivalent of 0.22 ± 0.19 D. The two devices were compared in terms of mean refractive prediction error (PE) was calculated as the difference between the postoperative and formula-predicted spherical equivalent using the IOL power implanted, as previously described [16]. The PE calculated for the PCI was been -0.69 ± 0.36 D, significantly greater compared to that of the SS-OCT (-0.24 ± 0.28 D; p<0.05). Results are summarized in Table 1.

**Table 1:** Mean axial length values from the two devices in the overall cohort and subgroups and statistical analysis.

	*N*	*PCI AL*	*SS-OCT AL*	*p-value*	*AL measurement errors (N)*
		*mean±SD; range (mm)*	*mean±SD; range (mm)*		
All patients	49	23.73 ± 0.98;	23.75 ± 0.97;	0.89*	PCI = 8; SS-OCT = 2
		21.56–26.44	21.98–26.45		
• ERM Foveoschisis	9	23.47 ± 1.24;	23.46 ± 1.25;	0.98^†^	Both devices = 0
		22.44–26.44	22.39–26.45		
• Pseudohole	4	23.94 ± 1.04;	23.93 ± 1.03;	0.99^†^	Both devices = 0
		23.31–25.49	23.27–25.46		
• ERM (all)	36	23.77 ± 0.93;	23.81 ± 0.90;	0.84*	PCI = 8; SS-OCT = 2
		21.56–25.96	21.98–26.04		
○ ERM stage 1	10	23.77 ± 0.83;	23.78 ± 0.81;	0.99^†^	Both devices = 0
		22.62–25.01	22.67–25.03		
○ ERM stage 2	9	23.80 ± 0.80;	23.80 ± 0.81;	0.99^†^	Both devices = 0
		22.41–24.92	22.44–24.92		
○ ERM stage 3	9	23.62 ± 1.13;	23.77 ± 1.06;	0.78^†^	PCI = 3; SS-OCT = 1
		21.56–25.45	21.98–25.47		
○ ERM stage 4	8	23.89 ± 1.07;	23.91 ± 1.06;	0.97^†^	PCI = 5; SS-OCT = 1
		23.00–25.96	23.04–26.04		

PCI: partial coherence interferometry; AL: axial length; SD: standard deviation; SS-OCT: swept-source optical coherence tomography; ERM: epiretinal membrane

*: Wilcoxon signed-rank test

^†^: paired t-test.

## Discussion

In this prospective cohort study, we evaluated the accuracy of AL measurements using two different devices in a cohort of patients presenting age-related cataract and ERM needing combined cataract and vitreoretinal surgery.

Axial length measurement errors were defined on the basis of AL measurement and retinal peak(s) appearance (Fig 1). Typically, AL measurement errors are characterized by a double-peak appearance of the retinal wave, with an apparently shorter AL and a consequent myopic shift in case of IOL implantation based on that data.

Overall, no significant differences in AL measurements emerged among the two devices (p = 0.89) as well as in each subgroup (Table 1).

The SS-OCT device presents a high wavelength (i.e. over 1000 nm), that provides a great tissue penetration and in ocular biometry it demonstrated a high efficiency in both healthy and age-related cataract eyes [17–19]. However, no previous literature compared PCI and SS-OCT devices in patients with VRI disorders (e.g. ERM or VMT), where AL measurement errors can occur, due to the retinal and epiretinal alterations [20].

Previous studies used PCI devices to study AL measurements in patients with VRI disorders, presenting different results. Kojima et al observed the presence of a double peak using a PCI device in the 35% of patients presenting an ERM, greater than macular oedema (20.5%) and macular hole (4.2%) [8]. The authors reported a significative positive correlation between interpeak distance and central retinal thickness and concluded that, in presence of a double signal within the retinal peak, the second peak (representing the RPE) should be considered for a correct AL calculation. Kim et al, in a cohort of eyes with cataract and ERM found a postoperative myopic shift using both ultrasounds and PCI [21]. Similar results were observed by Kovacs et al, who proposed, in patients with ERM, an adjustment of the AL measured with ultrasounds considering the central retinal thickness derived with the OCT [22].

On the other hand, Van der Geest et al, using a PCI device to calculate IOL power in patients undergoing phacovitrectomy for VRI disorders, found no significant differences in post-operative refractive outcomes as compared to a control group (phacoemulsification only, no VRI disorders) [23]. More recently, Vounotrypidis et al, compared the IOL Master 500 with the IOL Master 700 in the evaluation of the biometric measurements and predictive refractive accuracy in patients affected by age related cataract and VRI disorders [24]. The authors found significant differences between the devices in terms of prediction error within 0.5 dioptres and mean absolute error, both in favour of the IOL Master 700 [24].

In this study, a low rate of AL measurement errors was observed with both devices, being significantly lower for the SS-OCT (2/49, 4.1%) as compared to the PCI (8/49, 16.3%), p = 0.045. Interestingly, the AL measurement errors were all observed, for both devices, in eyes with ERM stages 3 and 4 (i.e. with ectopic inner foveal layers) with, respectively, one and one error for the SS-OCT and three and five errors for the PCI device (p = 0.02) (Figs 2 and 3). These findings resulted in a clinically relevant difference in terms of refractive PE, that would have brought to a significantly greater mean myopic shift of 0.69 D with the PCI compared to 0.24 D for the SS-OCT device (p<0.05).The cause of AL measurement errors in patients with severe ERM could be related to: (1) a thick ERM / posterior hyaloid (that being highly reflective accentuate the vitreo-retinal interface), (2) the attenuation of the RPE reflectivity caused by the ERM itself, and (3) by a thicker retina and the presence of ectopic inner foveal layers [8, 25].

**Fig 4:**
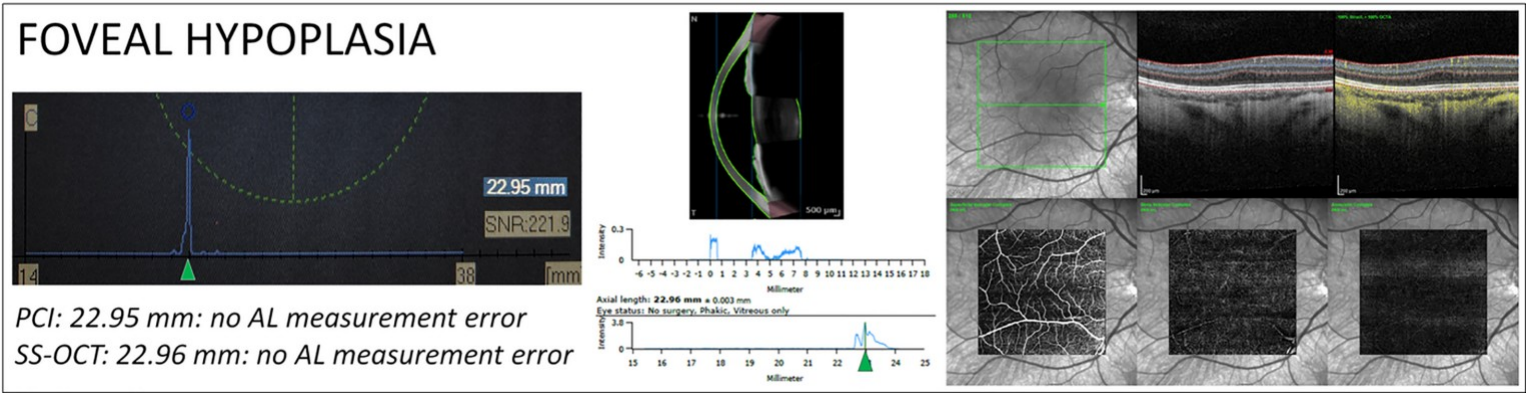
AL measurements in a patient with foveal hypoplasia. Biometric outputs, OCT and OCT-angiography (OCT-A) from a young patient with foveal hypoplasia. Left part: PCI biometric output. The measured mean AL is 22.95 mm. Only one peak is visible (green arrowhead). Middle part: SS-OCT biometric output (bottom). The measured mean AL is 22.96 mm. A moderate double peak is visible, with the first likely representing the vitreoretinal interface and the second the RPE (green arrowhead). Right part: Upper row: Infrared image (left), structural OCT B-scan passing through the fovea (middle) and structural B-scan with flow overlay (yellow dots). Lower row: enface OCT-A scans from the superficial vascular complex (left), deep vascular complex (middle) and avascular complex (right). AL measurements from both devices were considered correct.

However, a thick ERM / posterior hyaloid could be found in ERM stages 1 and 2, where no AL measurement errors were observed and the presence of intraretinal cysts, as well, could be observed in all ERM stages, in ERM with foveoschisis and in other retinal diseases, with a lower incidence of AL measurement errors [8, 26]. Finally, the presence of inner foveal layers is also observed in cases of foveal dysgenesis (e.g. foveal aplasia, Fig 4) and may persist after ERM+ILM peeling, with no reported cases of AL measurement errors.

As previously reported, ERM stage 3 and 4, presenting a poorer surgical prognosis, are characterized by greater retinal thickness, ectopic inner foveal layers and a consequent lower visibility of the outer retinal structures [13, 27, 28]. We can hypothesize that the coexistence of all these features is at the basis of the AL measurement errors in our cohort of patients with ERM stage 3 and 4.

Although a significantly lower number of AL measurement errors was observed using the SS-OCT device due to its intrinsic characteristics, the risk of a significant postoperative myopic shift in patients with severe ERM remains remarkable. In such cases, a differed approach with the vitreoretinal surgery performed before the cataract surgery may reduce this by removing the epiretinal hyperreflective material and inducing a normalization of the retinal thickness. However, this approach unlikely resolves the inner retinal layers ectopia and, on the other hand, presents the disadvantages of a two-step surgery [3, 29–31].

This study has some limitations. First, our results should be interpreted with caution as the sample size is small, especially for subgroup analysis; this is particularly evident in the subcohort of pseudohole patients; nevertheless, the main finding of the study was the significantly higher number of axial length measurement errors with the PCI in ERM patients groups 3 and 4, which was observed on a relatively larger sample size. Further studies on bigger cohorts are needed to support our data. Second, a single valid exam was performed with each machine, therefore impeding an evaluation of data repeatability. However, the repeatability of each instrument was already tested in previous studies [9, 10]. Third, although the aim of our study was to compare the AL measurements using two different technologies, we do not have postoperative data on patients’ refraction, that would have been useful in quantifying the refractive outcomes.

In conclusion, the PCI and SS-OCT devices present great accuracy in the AL calculation in patients with age-related cataract and ERM. In a limited subgroup of patients presenting more severe ERM, characterized by inner foveal layer ectopia, AL measurement errors were observed with both devices and were significantly less frequent with the SS-OCT. The choice of the IOL in such cases should be cautiously performed by looking at the biometric graphs, to avoid unexpected postoperative refractive outcomes.

## Supporting information

S1 Dataset mean Axial Length (AL) values (mm) obtained for each patient using the Partial Coherence Interferometry (PCI) and the Swept-Source OCT (SS-OCT) device. Patients data are sequentially reported and grouped on the basis of their vitreoretinal interface disorder in: epiretinal membrane (ERM, stages 1 to 4), macular pseudohole and ERM with foveoschisis.
(XLSX)
